# Editorial: Prostate cancer genomics: Application at different stages of a patient’s journey

**DOI:** 10.3389/fonc.2022.1101678

**Published:** 2022-11-29

**Authors:** Darren M. C. Poon, Arun Azad, Elena Castro, Ravindran Kanesvaran, Oliver Sartor

**Affiliations:** ^1^ Department of Clinical Oncology, State Key Laboratory of Translational Oncology, Sir YK Pao Centre for Cancer, Hong Kong Cancer Institute, The Chinese University of Hong Kong, Hong Kong, Hong Kong SAR, China; ^2^ Comprehensive Oncology Centre, Hong Kong Sanatorium and Hospital, Hong Kong, Hong Kong SAR, China; ^3^ Department of Medical Oncology, Peter MacCallum Cancer Centre, Melbourne, VIC, Australia; ^4^ Genitourinary Cancer Translational Research Group, Institute of Biomedical Research in Málaga, Málaga, Spain; ^5^ Division of Medical Oncology, National Cancer Centre, Singapore, Singapore; ^6^ Tulane Cancer Center, Tulane University Medical School, New Orleans, LA, United States

**Keywords:** *BRCA*, ctDNA, genetic testing, histone lysine methylation, microenvironment, molecular targeted therapy, prostate cancer

Over the past decade, genetic testing in prostate cancer (PCa) has become increasingly important, both in research and clinical application. Studies on potential clinical indications have expanded across the entire disease spectrum, from risk prediction in mutation carriers and patients with localized disease, to treatment planning and selection in those with advanced disease stages. This Research Topic is aimed at facilitating the exchange of scientific knowledge in PCa genomics and the implementation of individualized management of PCa patients across different disease stages, with the ultimate goal of optimizing patient care. This series includes four articles, which cover the relationship between the PCa microenvironment and oncological outcomes, and the broader role of genetic testing in PCa management.

To facilitate risk prediction and treatment selection, understanding the molecular mechanisms associated with PCa progression is crucial. Epigenetic reprogramming through dysregulated histone lysine methylation (HLM) is considered to accelerate the development of PCa. In a comprehensive analysis of 91 HLM regulators acknowledged to influence PCa, Quan et al. used bioinformatics and clinical tumor tissues to demonstrate that three HLM regulators (EZH2, NSD2, and KMT5C) were consistently upregulated in advanced PCa (Gleason score > 7, pathological T stage 3, and TP53 mutation). They also found that several HLM phenotype-related genes were predictive of tumor aggressiveness, recurrence-free survival, and responses to novel therapies with immune checkpoint inhibitors, which may provide a new therapeutic opportunity for patients with advanced PCa. Another study (Swami et al.) investigated the association between genomic landscapes and responsiveness to poly (ADP-ribose) polymerase (PARP) inhibitors, a recently approved therapy for *BRCA*-mutated metastatic castration-resistant PCa. Their analysis of co-segregating genes among men with advanced PCa who harbored *BRCA1* and/or *BRCA2* alterations demonstrated that, in contrast to *BRCA1*, *BRCA2* had no significant associations with any gene, which implies its status of main driver mutation and thus higher sensitivity to PARP inhibition. These studies may enhance the understanding of prognostic factors, optimize treatment selection, and encourage further evaluation of the tumor genomic microenvironment in patients with advanced PCa.

With the development of research on prognostic and therapeutic biomarkers, the clinical value of genetic testing in the management of PCa is increasingly recognized. Representatives of the Hong Kong Urological Association and the Hong Kong Society of Uro-Oncology established a set of consensus statements (Chiu et al) to address the indications, methods, and therapeutic implications of genetic testing in patients with PCa across different disease stages, with the aim of providing guidance to clinicians in Hong Kong, and possibly across the Asia-Pacific region, regarding who and what to test for in selected populations. While the methods and techniques of genetic testing are ever-changing, analysis of circulating tumor DNA (ctDNA) from blood plasma remains an emerging and convenient approach, particularly when tumor tissue is not readily available. Kwan et al. reviewed the current and potential applications for ctDNA analysis in metastatic PCa, which include prognosis prediction, treatment planning, and characterization of treatment resistance. They also discussed the possible limitations in interpreting ctDNA findings, particularly false-negative results that occur with low tumor content, and the approaches to optimize assay design, including correction for clonal hematopoiesis of indeterminate potential and germline variants. With the advancement of assay technology and accessibility, the role of genetic testing in the management of PCa continues to evolve, potentially informing the implementation of precision medicine initiatives and treatment individualization.

Further research on PCa genomics is expected and warranted. Molecular mechanisms of mutations and actionable biomarkers in specific patient populations are among the fields of clinical significance. Randomized trials and real-world studies alike are eagerly awaited to provide insights on how novel diagnostic, therapeutic, and management approaches based on genetic informatics could improve clinical outcomes in patients with both localized and advanced PCa.

## Author contributions

This manuscript was drafted and critically reviewed by DP, AA, EC, RK, and OS. All authors contributed to the article and approved the submitted version.

## Conflict of interest

The authors declare that the research was conducted in the absence of any commercial or financial relationships that could be construed as a potential conflict of interest.

## Publisher’s note

All claims expressed in this article are solely those of the authors and do not necessarily represent those of their affiliated organizations, or those of the publisher, the editors and the reviewers. Any product that may be evaluated in this article, or claim that may be made by its manufacturer, is not guaranteed or endorsed by the publisher.

